# Detection of Mycotoxigenic Fungi and Residual Mycotoxins in Cannabis Buds Following Gamma Irradiation

**DOI:** 10.3390/toxins17110528

**Published:** 2025-10-28

**Authors:** Mamta Rani, Mohammad Jamil Kaddoura, Jamil Samsatly, Guy Chamberland, Suha Jabaji, Saji George

**Affiliations:** 1 Department of Food Science and Agricultural Chemistry, McGill University, Montreal, QC H9X 3V9, Canada; mohammad.kaddoura@mail.mcgill.ca (M.J.K.);; 2 BioSun Solutions, Biotechnology Company, Chambly, QC J3L 4V2, Canada; jsamsatly@biosunsolutions.com (J.S.);

**Keywords:** cannabis, microbial contamination, mycotoxigenic fungi, mycotoxins, *Aspergillus*, *Fusarium*, *Penicillium*, gamma irradiation, PCR/qPCR

## Abstract

Cannabis plants are susceptible to microbial contamination, including fungi capable of producing harmful mycotoxins. The presence of these toxins in cannabis products poses serious health risks, especially when used for medical purposes in immunocompromised people. This study evaluated the presence of fungi and mycotoxins in dried cannabis buds following gamma irradiation, using culture-based techniques, PCR/qPCR, and ELISA. Irradiation significantly reduced fungal and bacterial loads, eliminating culturable bacteria but did not achieve complete sterilization. Viable spores of toxigenic fungal genera, such as *Aspergillus*, *Penicillium*, and *Fusarium,* persisted. Sequencing of ITS amplicons revealed dominant mycotoxigenic fungi in non-irradiated (NR), irradiated (IR) and licensed producer (LP) samples, while next-generation sequencing (NGS) revealed additional non-culturable toxigenic species. PCR/qPCR detected biosynthetic genes for aflatoxins, trichothecenes, ochratoxins, and deoxynivalenol across all samples, with gene copy numbers remaining stable post-irradiation, suggesting DNA damage without full degradation. ELISA confirmed aflatoxin, ochratoxin, DON, and T2 toxins in both IR and LP samples at variable concentrations. While LP samples showed lower microbial counts and gene abundance, residual DNA and toxins were still detected. Our study shows that while irradiation decreases microbial loads, it does not completely remove toxigenic fungi or their metabolites. Ensuring the safety of cannabis products necessitates a multifaceted assessment that incorporates cultural, molecular, and immunological techniques, in parallel with more stringent microbial standards during production stage.

## 1. Introduction

Mycotoxins are harmful secondary metabolites produced by a wide range of toxigenic filamentous fungi whose presence in food and feed is recognized as a global concern due to their severe implications on human and animal health [1,2]. Consumption of these toxins through contaminated products can cause a variety of symptoms from acute such as vomiting and abdominal pain to the development of chronic conditions including immunosuppression, tumors, and even death [3,4,5,6]. The most notable causal fungi are members of the *Aspergillus*, *Fusarium*, and *Penicillium* genera, producing mycotoxins such as aflatoxins, ochratoxins, fumonisins, deoxynivalenol/nivalenol, and zearalenone [7,8,9].

While mycotoxins are most closely monitored in staple crops, they are also prevalent contaminants in herbal products, including cannabis. Cannabis inflorescences are highly susceptible to colonization by mycotoxin-producing fungi such as *Aspergillus*, *Penicillium*, and *Fusarium* due to their dense, nutrient-rich, moisture-retaining structure, and favorable post-harvest practices [10]. Like other plants, cannabis harbors fungal endophytes such as *Aspergillus* and *Penicillium*, which generally colonize plant tissues asymptomatically but can shift to pathogenic behavior under certain conditions or during plant stress [11,12,13]. Environmental stressors such as elevated humidity, plant senescence, or weakened plant defenses may activate these fungi, leading to opportunistic pathogenicity and mycotoxin biosynthesis. Moreover, inadequate post-harvest drying or storage can further exacerbate mycotoxin accumulation in cannabis buds, increasing health risks for consumers.

Fungal and bacterial contamination has been reported in more than 20% cannabis intended for medicinal use [14]. Microbial surveys have detected diverse contaminants, including *Aspergillus* spp. (*A. fumigatus*, *A. flavus*, *A. niger*, *A. repens*, *A. tamarii*), *Mucor*, *Penicillium*, *Candida*, and plant viruses [15,16]. This poses major health hazards, with heightened risk for immunocompromised populations such as cancer patients, transplant recipients, and individuals with HIV/AIDS. Multiple case reports link its use to pulmonary aspergillosis and other opportunistic infections. Examples include chronic necrotizing pulmonary aspergillosis in a 29-year-old man [17], invasive pulmonary aspergillosis in a cancer patient [18], and fatal aspergillosis in a lung transplant recipient exposed to marijuana smoke [19]. Such infections have also been reported in healthy users [20]. This is especially problematic, given that 70% of cannabis is consumed by smoking or vaporizing, hence directly delivering spores and toxins into the highly absorptive lung tissues [21]. These findings highlight the pressing need for comprehensive mycotoxin monitoring as part of standard safety protocols, thereby reducing health risks for consumers and especially for vulnerable populations.

Cannabis producers often employ heat or pasteurization methods to reduce microbial loads. While curing and heat-treating processes can reduce viable microbial count, these methods do not reliably eliminate spores, viral DNA, or microbial toxins [22,23]. Gamma radiation has been recommended as a sterilization technique because of its detrimental action on microorganisms without substantial effect on product quality. *Aspergillus* spores and toxins are particularly resistant and can withstand sterilization, processing, and extraction conditions. Consequently, routine testing for mycotoxigenic fungi has been recommended by the Cannabis Safety Institute. Fewer than 1% of cannabis studies have examined microbial contamination, and only 0.5% have addressed mycotoxins or spoilage organisms [14,24]. This underscores a critical knowledge gap and highlights the urgent need for developing improved detection methods to safeguard consumer health.

To address these concerns, this study applied three complementary approaches to assess fungal contamination and mycotoxin presence in cannabis buds: (1) culture-based methods to isolate and identify viable fungi, (2) PCR/qPCR to detect biosynthetic genes associated with major mycotoxins, and (3) ELISA to quantify toxin residues. The findings demonstrate that fungi and their metabolites can persist after gamma-irradiation, and that standard detection methods such as plating and ELISA may be insufficient to fully capture microbial and mycotoxin contamination in products labeled as “market safe”.

## 2. Results

### 2.1. Isolation and Identification of Microorganisms

A total of twelve cannabis samples (IR, NR, and LP1–LP10) were analyzed. Cultivation methods included greenhouses (*n* = 5), hybrid greenhouses (*n* = 3), and indoors (*n* = 4). Detailed sample profiles are provided in Table 1. Chemotypes were mostly THC-dominant (12–26%) with low CBD (0–2%). Myrcene, caryophyllene, limonene, and pinene were the most frequently detected terpenes.

#### 2.1.1. Culture-Based Methods to Enumerate Microbial Contamination

Morphological and microscopic examinations were used for the preliminary identification of fungal and bacterial cultures. From NR samples, 10 bacterial and 53 fungal strains were isolated using PDA, LBA, MEA, and DRBC. Of all isolates, 30 fungal isolates showing distinct colony features (e.g., morphology, color, and size) were selected for further genomic profiling. *Enterobacter hormaechei*, *Methylobacterium tardu*, and *Pseudomonas aeruginosa* were the predominant bacterial isolates. In contrast, in the case of IR, no bacterial growth was detected and 30 fungal strains were recovered.

The dilution plating assay was conducted on both selective and non-selective culture, with or without antibiotics. Colony forming units were enumerated after 48 h for bacteria and 120 h for fungi. In NR samples, the highest fungal abundance (TYM) was observed on PDA, reaching 5 × 10^4^ CFU.g^−1^, whereas IR samples yielded 3 × 10^4^ CFU.g^−1^. Bacterial counts in NR samples peaked at 7 × 10^3^ CFU.g^−1^, while no bacterial growth was observed in IR samples. Microbial load assessment was repeated at three time points, with consistent counts observed across replicates. Among LP samples, fungus CFUs were only detected in LP5 at 1.2 × 10^4^ CFU.g^−1^, while no growth was observed in the remaining samples. Bacterial growth was limited to LP1 and LP5 each showing approximately 2 × 10^3^ CFU.g^−1^ of cannabis tissue (Table S1, Figure S1).

After removal of duplicate colonies, 40 fungal isolates from NR and IR samples were Sanger sequenced for ITS regions, of which 26 strains were successfully identified (NR, *n* = 20; IR, *n =* 6). The identified taxa belonged to *Aspergillus* (*A. flavus*, *A. japonicus*, *A. ochraceus*, *A. sclerotiorum*, and *A. tamarii*), *Penicillium* (*P. commune*, *P. copticola*, *P. citrinum*, *P. simplicissimum*, and *P. griseofulvum*), *Cladosporium* (*C. tenuissimum* and *C. oxysporum*), and *Fusarium* (*F. proliferatum*). In NR samples, *Penicillium* (10 isolates) and *Aspergillus* (7 isolates) were most abundant, followed by *Cladosporium* and *Fusarium* (Table 2).

Several isolates are well-documented mycotoxin producers. In NR samples, *P. citrinum* (citrinin) and *P. simplicissimum* (verruculogen) were detected, while in IR samples, *P. griseofulvum* (patulin, griseofulvin) and *P. commune* (cyclopiazonic acid, roquefortine C). Within the *Aspergillus* genus, isolates included *A. japonicus* (alkaloids), *A. flavus* (aflatoxins), *A. ochraceus* (ochratoxins), *A. sclerotiorum* (penicillic acid and ochratoxins), and *A. tamarii* (cyclopiazonic acid). Among the LP samples, mycotoxigenic fungi belonging to *Aspergillus*, *Chaetomium*, and *Penicillium*, were detected in samples LP1, LP5, and LP7. Additionally, pathogenic bacteria *Methylobacterium tardum* and *P. aeruginosa* were recovered from LP1 and LP5, respectively. Details of identified taxa and the relevant associated toxins are presented in Table 3.

#### 2.1.2. Identification of Microbial Communities by NGS

NGS analysis of NR and IR samples identified *A. ochraceous*, *P. copticola*, and *P. simplicissimum* in both treatments. Additional species not recovered by culture-based methods or Sanger sequencing were detected, including *Diaporthe caulivora*, *P. steckii* (produces citrinin), *P. olsonii*, *Sarocladium strictum*, *Cladosporium sphaerospermum*, *Trichoderma hamatum*, *Fusarium intricans*, and *Ophiosphaerella agrostidis*. Taxonomic abundance was higher in NR compared to IR for most identified genera. Notably, *Penicillium copticola* (0.00284 in NR vs. 8.99 × 10^−5^ in IR), *P. simplicissimum* (0.00122 vs. 0.00025), *D. caulivora* (0.00063 vs. 2.40 × 10^−5^), *C. sphaerospermum* (7.79 × 10^−5^ vs. 3.60 × 10^−5^) and *A. ochraceus* (6.59 × 10^−5^ vs. 1.20 × 10^−5^) were more abundant in NR samples (Table S2).

### 2.2. Detection of Mycotoxin-Related Genes Using Conventional PCR and Quantification Using qPCR

PCR screening with target-specific primers confirmed the presence of the selected 5 biosynthetic genes (*DON*, *Tri5*, *Tri6*, *PKS*, and *Nor-1*) in NR samples, indicating the presence of intact mycotoxin gene clusters. In LP1-LP10 samples, *PKS* was absent in LP6 and LP9, *Nor1* was absent in LP1 and LP9, *Tri5* was absent in LP4, and LP8-LP10, whereas *DON* and *Tri6* genes were detected in all samples. Following PCR confirmation, qPCR analysis was performed to quantify gene copy numbers and enable comparison across treatments. Standard curve slopes ranged from –3.00 to –3.34 and efficiencies between 85% to 100% (R^2^ > 0.99), within the acceptable range. However, DON quantification was excluded from analysis due to poor standard curve performance.

For NR samples, the average copy numbers were 8.0 × 10^7^ copies/g for *Nor1*, 4.83 × 10^8^ copies/g for *Tri5*, 2.11 × 10^9^ copies/g for *PKS*, and 2.07 × 10^11^ copies/g for *Tri6*. For IR samples, corresponding values were 7.1 × 10^7^ copies/g for *Nor1*, 4.65 × 10^8^ copies/g for *Tri5*, 1.69 × 10^9^ copies/g for *PKS*, and 6.57 × 10^10^ copies/g for *Tri6*. Among LP samples, *Nor1* was detected in all except LP1 and LP9, with copy numbers ranging between 8.7 × 10^4^ copies/g (LP3) and 1.7 × 10^5^ copies/g (LP6). *Tri5* was absent in LP4, LP9, and LP10 but present in other samples at 8.6 × 10^5^ to 7.0 × 10^6^ copies/g. *PKS* was detected in all samples except LP9, with values between 8.3 × 10^6^ (LP5) to 6.73 × 10^8^ (LP7) copies/g. Tri6 was consistently identified in all samples, ranging from 3.3 × 10^6^ (LP1) to 3.3 × 10^7^ copies/g.

Significant differences in copy numbers were observed between NR and IR samples (*p* > 0.05, Tuckey HSD). The highest copy number was recorded for *PKS* with comparable levels between NR and IR. Overall, *Nor1*, *Tri5*, *PKS*, and *Tri6* copy numbers were similar across treatments, while DON results were excluded due to unreliable standard curves. Average Cq values and copy numbers are presented in Table 4.

### 2.3. Detection of Mycotoxins by Enzyme-Linked Immunosorbent Assay (ELISA)

ELISA based detection was utilized for the confirmation of presence of detectable amount of prominent mycotoxins in these samples. Our investigations showed presence of Ochratoxin A in all samples, ranging from 0.7 ppb (LP3) to 3.8 ppb (LP5), with concentrations of 6.3 ppb in IR and 8.1 ppb in NR. Aflatoxins were also present across all samples varying from 1.2 ppb (LP4) to 3.1 ppb (LP10), with IR and NR samples showing 4.9 ppb and 5.7 ppb, respectively. DON concentrations ranged from 0.02 ppm (LP4) to 0.81 ppm (LP2), while IR and NR samples had comparable levels 2.05 and 2.08 ppm. T2 toxins exhibited the widest range, from 5 ppb (LP4) to 23.8 ppb (LP10), with 10.8 ppb in IR and 15.1 ppb in NR. The highest concentration overall was recorded for T2 toxin in LP10 (23.8 ppb). ELISA detected all four toxins in LP samples regardless of visible microbial contamination (Table 5).

## 3. Discussion

Following legalization in Canada in 2018, cannabis consumption has increased markedly. Regulatory frameworks attempt to address presence of biohazards by establishing microbial and chemical safety standards for both medicinal and recreational cannabis products. Total yeasts and molds (TYM) are typically quantified as colony-forming units per gram (CFU.g^−1^) of dried inflorescences. These limits vary internationally from 1000–10,000 CFU.g^−1^, and in certain jurisdictions as high as 50,000–100,000 CFU.g^−1^. The regulatory threshold in Canada is set at 50,000 CFU.g^−1^ by Health Canada [40]. In contrast, threshold limits in the United States differ by state, reflecting the absence of a unified national standard. Canadian mycotoxin regulatory limits align with international guidelines established by the Joint FAO/WHO Expert Committee on Food Additives (JECFA) and with the maximum limits set by the European commission under Regulation (EU) 2023/915 [41,42]. The combined concentration of aflatoxins (B1, B2, G1, and G2) and ochratoxin A must not exceed 20 μg/kg. These toxins are the primary focus of regulations due to their potent carcinogenicity and nephrotoxicity.

Gamma irradiation substantially reduced fungal and bacterial loads in cannabis buds but failed to achieve complete sterilization, defined here as the total absence of viable microorganisms. Although all IR samples complied with Health Canada’s regulatory threshold <50,000 CFU.g^−1^, culture-based assays still recovered fungal colonies from treated material. These include members of genera known to contain mycotoxigenic and pathogenic species such as *Aspergillus*, *Penicillium*, and *Fusarium*. Notably, *F. proliferatum* was only detected in NR samples, whereas no bacterial growth was observed in IR material, consistent with prior reports that gamma irradiation is effective against vegetative bacterial cells [22,23].

The persistence of *Aspergillus* spores and their associated mycotoxins in treated samples is consistent with prior reports documenting their resistance to gamma irradiation [10,43,44]. These spores possess a multilayered cell wall structure enriched with melanin, which acts both as a physical barrier and as a scavenger of reactive oxygen species, protecting genomic integrity from radiation-induced oxidative damage [45,46,47]. This structural resilience likely contributes to the reduced efficacy of irradiation against *Aspergillus* in cannabis products.

Cultural-based methods remain valuable for detecting viable microorganisms that may pose immediate health risks with an increased risk upon inhalation, particularly for immunocompromised consumers. However, these methods are generally time-intensive, require trained personnel, and favor fast-growing molds while underestimating slow-growing or non-culturable species. Molecular methods confirmed and expanded upon the culture-based findings. Sanger sequencing of cultured fungal isolates revealed the dominance of *Penicillium* and *Aspergillus* in NR samples, with additional identification of *Cladosporium* and *Fusarium*. Species confirmed in both NR and IR samples included *P. citrinum*, *P. simplicissimum*, and *A. flavus*, all of which are known producers of citrinin, verruculogen, and aflatoxins, respectively. *P. copticola*, a species previously associated with *Cannabis sativa*, was also recovered and is known to produce patulin and ochratoxins [13]. Depending on the exposure intensity, these toxins have the potential for gastrointestinal toxicity, nephrotoxicity, immunotoxicity, genotoxicity, and other systemic effects. *P. griseofulvum* and *P. commune*, both capable of producing patulin, griseofulvin, cyclopiazonic acid, and other neurotoxic metabolites were detected in IR samples (Table 3). This indicates that while irradiation reduced microbial diversity and occurrence, it did not eliminate all toxigenic species. LP cannabis products showed lower contamination overall. Only three of ten products (LP1, LP5, and LP7) contained detectable mycotoxigenic fungi, while the rest showed no microbial growth. Nonetheless, pathogenic bacteria such as *Methylobacterium tardium*, *P. aerginosa* were detected in LP1 and LP5. These results suggest that while licensed cannabis products undergo more rigorous processing, contamination by opportunistic microbes may still occur.

NGS revealed a broader fungal diversity than detected by either culture or Sanger sequencing. Both NR and IR samples contained *A. ochraceus*, *P. copticola*, and *P. simplicissimum*, but additional taxa were identified exclusively through NGS, including *Diaporthe caulivora*, *P. steckii*, *Sarocladium strictum*, *Cladosporium sphaerospermum*, *Trichoderma hamatum*, *Fusarium intricans*, and *Ophiosphaerella agrostidis*. Several of these including *P. steckii* and *D. caulivora*, are associated with mycotoxin production and crop pathogenesis. These observations support the use of NGS to uncover microbiological threats that evade conventional methods, particularly non-culturable or low abundance organisms of toxicological concern.

PCR and qPCR results confirmed the presence of mycotoxins biosynthetic genes in all sample types. *PKS* and *Tri6* were consistently amplified in nearly all products, whereas *Nor1* and *Tri5* were variably absent or detected at low abundance (Cq > 32). Although gene presence does not equate to active toxin production, the consistent detection in IR and LP samples raises safety concerns. Gene copy numbers measured by qPCR did not significantly differ between NR and IR groups, suggesting that gamma irradiation reduced DNA integrity but did not fully degrade fungal genomes.

ELISA confirmed the presence of major mycotoxins including Ochratoxin A, aflatoxins, DON, and T2 across NR, IR, and LP samples. Notably LP samples tested positive for one or more mycotoxins despite passing culture-based microbial testing, highlighting the persistence of toxin residues even in “clean” market products. The highest concentration of T2 toxins was recorded in LP10. These findings align with previous reports supporting ELISA as a reliable tool for rapid mycotoxin screening [48]. However, the requirement for immunoaffinity cleanup to minimize matrix effects remains a limitation for routine testing.

Taken together, these results demonstrate that gamma irradiation reduces overall microbial load but does not reliably eliminate mycotoxigenic fungi, their biosynthetic DNA, or residual mycotoxins. This persistence poses a credible health risk, particularly for immunocompromised users of medical cannabis. Several case reports have linked cannabis use to pulmonary aspergillosis and other fungal infections. More recently, genome sequencing identified *Cryptococcus neoformans*, a major opportunistic pathogen, in medical cannabis that was genetically identical to isolates recovered from a patient with multiple myeloma who developed cryptococcal meningitis and subsequently died despite treatment [49]. These clinical observations emphasize the gap between regulatory compliance and actual consumer safety.

From a regulatory perspective, reliance solely on CFU-based testing is inadequate. Total yeast and mold limits vary widely across jurisdictions, ranging from 10^3^ to 10^5^ CFU.g^−1^, complicating risk assessment. The presence of fungal DNA and mycotoxins in LP samples that complied with microbial standards underscores the need for diagnostic approaches that integrate culture-based, molecular, and immunoassay methods. A zero-tolerance threshold (<10 CFU.g^−1^) for high-risk fungi such as *Aspergillus* and *Fusarium*, as practiced in some parts of the industry may offer better protection for vulnerable population. Ultimately, an integrated testing framework is required to assess the microbiological and toxicological risk. As the cannabis market expands to include both recreational and medical users, harmonized policies are needed to ensure the safety of all consumers.

## 4. Conclusions

This study provides comprehensive evidence that gamma irradiation, while effective in reducing viable microbial loads in cannabis products, is insufficient for eliminating toxigenic fungi, their biosynthetic genes, or residual mycotoxins. Through the integration of culture-based, molecular, and immunoassay techniques, we demonstrate that cannabis products passing the standard CFU-based testing may still harbor microbial threats with clinical relevance. Since gamma irradiation is insufficient for eliminating mycotoxins in cannabis buds, contamination must be addressed proactively across the supply chain. This requires cultivating plants under conditions that inhibit toxigenic fungal growth during production (e.g., using biocontrol agents), as well as ensuring proper storage and handling practices post-harvest. As the cannabis industry evolves under expanding legalization, microbial testing must move towards integrated risk-based frameworks that consider microbial viability, toxin persistence, and host vulnerability. A key limitation of this study is the small sample size, which reflects the practical challenges associated with obtaining cannabis samples for research under Canada’s stringent production and regulatory restrictions. Nonetheless, the sample quantity (100 g) was sufficient to conduct all planned experiments. All experiments were conducted in triplicate and repeated to confirm statistical reliability and reproducibility. While this limitation may constrain the generalizability of the findings, the results underscore the urgent need for proactive contamination control throughout the supply chain. Future regulations should prioritize comprehensive risk surveillance rather than presumed safety based on low CFU counts while investing in scalable, cost-effective detection protocols. Addressing these gaps will be critical to ensuring cannabis product safety for both recreational and medically vulnerable populations.

## 5. Materials and Methods

### 5.1. Sample Collection and Preparation

Two dried cannabis bud samples (100 g each) of the *Phytopain* cultivar, designated as non-irradiated (NR) and irradiated (IR), were provided by the Research and Development Department of a licensed producer (LP) in Quebec, Canada. In accordance with standard industry practice in Canada, the LP outsourced the irradiation process to a certified third-party facility approved by Health Canada. The specific facility and exact gamma irradiation dose were not disclosed; however, treatment was conducted within the standard 10–15 kGy range used for microbial decontamination of medicinal cannabis products. Ten commercial cannabis bud samples (LP1-LP10) were purchased from Société Québécoise du Cannabis (SQDC) retail outlets (Table 1). Samples were stored under controlled conditions and handled exclusively in a sterile environment. Experimental work was conducted at Macdonald Campus of McGill University (Sainte-Anne-de-Bellevue, QC, Canada).

### 5.2. Culture Media and Chemicals

Dichloran-rose Bengal-chloramphenicol agar (DRBC) was obtained from Difco Laboratories (Detroit, MI, USA). Potato Dextrose Agar (PDA), Malt Extract Agar (MEA), Nutrient Agar (NA), Luria-Bertani Agar (LBA), Cetyltrimethylammonium bromide (CTAB), and other common laboratory solvents were purchased from Fisher Scientific (Waltham, MA, USA). Antibiotics (kanamycin, rifampicin, and tetracycline), fungicide (benomyl), and miticide (ivermectin) were obtained from Sigma-Aldrich (St. Louis, MO, USA). Cellophane membranes (500 PUT) were purchased from UCB (North Augusta, SC, USA).

### 5.3. Molecular Biology Reagents and Sequencing

Primers and probes were synthesized by Integrated DNA Technologies (IDT, Coralville, IA, USA). Conventional PCR was performed using Taq polymerase PCR kits (Bio-Rad, Hercules, CA, USA), Phusion^®^ High-Fidelity PCR Master Mix (New England Biolabs, Ipswich, MA, USA), and PCR purification kit and pDrive cloning vectors (Qiagen, Hilden, Germany). qPCR assays were carried out with TaqMan^®^ Fast Advanced Master Mix (Thermo Fisher Scientific, Wilmington, DE, USA). Sequencing libraries were prepared with NEBNext^®^ Ultra™ DNA Library Prep Kit (New England Biolabs, Ipswich, MA, USA) and sequenced on an Illumina platform (Illumina, San Diego, CA, USA).

Conventional PCR was run on a T100 Thermal Cycler (Bio-Rad, Hercules, CA, USA), and qPCR was performed on an Mx3000P QPCR System (Agilent Technologies, Santa Clara, CA, USA). DNA quality and concentration were measured using a Nanodrop ND1000 spectrophotometer and Qubit fluorometer (Thermo Fisher Scientific, Wilmington, DE, USA). Sanger and next-generation sequencing (NGS) were carried out at the McGill Genome Center (Montreal, QC, Canada).

### 5.4. Enzyme-Linked Immunosorbent Assay (ELISA) Reagents

Commercial ELISA kits and Immunoaffinity columns for mycotoxin detection were obtained from Romer Labs (Getzersdorf, Austria). Pure standards for method validation were purchased from Sigma-Aldrich (St. Louis, MO, USA).

### 5.5. Isolation and Identification of Microorganisms

#### 5.5.1. Culture-Based Methods

##### Direct Plating Method

Each of the NR, IR, and LP1–LP10 dried flower samples (100 mg) was plated on DRBC, PDA, and LBA media. For bacterial isolation, the plates were supplemented with benomyl fungicide to suppress fungal growth. While for fungal isolation, the plates were amended with kanamycin, rifampicin, tetracycline, and ivermectin. Multiple culture media were used to accommodate different growth requirements and improve recovery of diverse microbial taxa. Single colonies were purified by repeated sub-culturing to ensure the purity of the strains. Fungal strains were identified based on both macroscopic and microscopic characteristics. All pure bacterial and fungal isolates were preserved as glycerol stocks at −80 °C for further use.

##### Dilution Plating Method

Each of the NR, IR, and IR lots, as well as LP1–LP10 dried flower samples (100 mg), was suspended in 1 mL of sterile distilled water (SDW) 1:10 *w*/*v* and agitated for 3 min at room temperature. Serial dilutions 10^−1^–10^−5^ were plated on DRBC, PDA, LBA, with or without antibiotics, and incubated at 28 °C. For bacterial isolation, CFUs were counted after 48 h, while for fungal isolation, CFUs were counted after 120 h. The complete procedure was repeated every 2 months to monitor microbial recovery.

#### 5.5.2. Identification of Isolates by ITS and 16S Sequencing

Fungal isolates were grown on PDA covered with cellophane membranes at 24 °C for 2 weeks. Mycelia were harvested, flash frozen in liquid nitrogen, and stored at −80 °C. DNA was extracted using CTAB and purified using the phenol-chloroform extraction. The ITS region was amplified with universal fungal primers ITS1 (5′-CTTGGTCATTTAGAGGAAGTAA-3′) and ITS4 (5′-TCCTCCGCTTATTGATATGC-3′) using 40 ng of genomic DNA for 50 μL reaction in total. PCR cycling conditions involved 35 cycles at 58 °C annealing temperature. For bacterial identification, the 16S rRNA region was amplified using universal bacterial primers 27F (5′-AGAGTTTGATCCTGGCTCAG-3′) and 534R (5′-ATTACCGCGGCTGCTGG-3′). PCR cycling conditions involved 35 cycles at 63 °C annealing temperature. Fungal and bacterial PCRs were performed using a PCR kit (Bio-Rad, Hercules, CA, USA). A no-template control (NTC) and positive control (PC) were included with each reaction. Amplified PCR products were confirmed on a 1% agarose gel. PCR products were sent for Sanger sequencing at the McGill Genome Center. Sequences were identified using BLAST searches against the National Center for Biotechnology Information (NCBI): (https://www.ncbi.nlm.nih.gov, accessed on 26 March 2021) database.

#### 5.5.3. Identification of Microbial Communities by NGS

To complement isolation sequencing, NGS was performed on NR and IR samples to identify microorganisms that were not recovered by culture-dependent methods. Genomic DNA was extracted using a modified CTAB method [50,51], and quality was assessed on a 1% agarose gel. DNA was diluted to 1 ng/µL in SDW. Amplicons targeting the 16S rRNA (V4: 515F-806R), 18S rRNA (V4: 528F-706R), and ITS (ITS1/ITS2; Arc V4) were generated using barcoded primers. PCR amplification was performed using Phusion^®^ High-Fidelity PCR Master Mix, and products were verified on 2% agarose gels. Amplicons of expected size 400–450 bp were pooled at equimolar concentrations, purified with the Qiagen Gel Extraction Kit. Sequencing libraries were prepared with the NEBNext^®^ Ultra™ DNA Library Prep Kit and were quantified using Qubit and qPCR and sequenced on an Illumina platform.

Paired-end reads were demultiplexed, trimmed, and merged with FLASH (v1.2.7). Quality filtering was performed with QIME (v1.7.0), and chimeric sequences were removed using UCHIME (v4.2) against the gold database [52]. Operational Taxonomic Units (OTUs) were clustered at 97% similarity using UPARSE (v7.0.1001) and taxonomically assigned in Mothur (v1.44.3) against the SILVA SSU rRNA database (confidence threshold 0.8–1.0). Phylogenetic relationships were inferred using MUSCLE (v3.8.31). OTUs abundance was normalized to the lowest sequences depth, and alpha- and beta- diversity analyses were conducted on the normalized data set using FLASH (V1.2.7).

### 5.6. Detection and Quantification of Mycotoxigenic Fungal Genes

Cannabis bud samples were pulverized into a fine powder in liquid nitrogen, and DNA was extracted using the modified CTAB method. Extracted DNA was eluted in SDW and stored at −20 °C until use. DNA concentration and quality were assessed using a Nanodrop ND1000 spectrophotometer and on 1% agarose gels.

#### 5.6.1. Detection of Mycotoxins Related Genes Using Conventional PCR

Primers targeting aflatoxin (*Nor1*), ochratoxin (*Pks*), and three major trichothecenes (*Tri5*, *Tri13*, and *Tri6*) were designed based on literature (Table S3). Primers were checked for secondary structures and specificity was verified using BLAST (NCBI). Positive control genomic DNA was obtained from reference isolates *A. flavus* -PF4 and -PF41 isolated from IR and NR samples, *A. ochraceus* -V5AB isolated from the production facility of a cannabis licensee producer, and *F. graminearum* -Fl1sta, -Fl1b, -Fl1stb1, -F12sta, and -F12stc isolated from cannabis flowers from the LP. All isolated were confirmed by Sanger sequencing prior to use.

Conventional PCR was performed on 20 ng of DNA using the Taq polymerase PCR kit. PCR cycling conditions consisted of 35 cycles with annealing temperature at 58 °C. A no-template control and the reference isolate were included with each reaction. PCR products were visualized on 1% agarose gels and representative PCR products were sequenced at Genome Quebec (McGill University, Montreal, QC, Canada). Sequences were identified using BLAST (NCBI).

#### 5.6.2. Quantification of Mycotoxins Related Genes Using qPCR

##### Primer and Probe Design

Primer and probes targeting *Nor1*, *Pks*, *Tri5*, and *Tri13* were designed from NCBI GenBank sequences (AY371490.1, AY320069, AF336366, AY064209) and synthesized with PrimerQuest^®^ (Integrated DNA Technologies, Inc. [IDT], Coralville, IA, USA) (Table 6). OligoAnalyzer (IDT, Coralville, IA, USA) and BLAST were used to confirm specificity. Genomic DNA extracted from all twelve samples was tested.

##### Absolute Quantification of Mycotoxin Genes

PCR products of the target genes were purified using Qiagen PCR purification kit and cloned their pDrive vector following the manufacturer’s protocol. Successful gene insertion was confirmed using respective gene primers and sent for sequencing at Genome Quebec then verified by to BLAST (NCBI).

Plasmid DNA was 10-fold serially diluted (from 10^−1^ to 10^−10^) and used to generate the standard curves and the Cq values were plotted against the logarithm of the copy number of the target gene. The copies numbers were calculated using the formula:

Number of copies = [Amount of DNA (ng) × (6.022 × 10^3^)]/[Length of DNA template (bp) × 1 × 10^9^ × 660]

The reliability of the standard curves was assessed based on the slope, correlation coefficient (R^2^), and amplification efficiency, where efficiency (E) was calculated based on the equation:

E = 10^−1/S −1^, with S representing the slope of the regression Cq versus log copy number. Acceptable criteria were a slope between −3.0 and −3.34 and an amplification efficiency between 100 ± 10%.

qPCR was performed using TaqMan^®^ Fast Advanced Master Mix, which contained ROX™ Passive Reference dye as internal control. Reaction mixtures contained: 0.2 µM primers, 0.1 µM probe, 2X Master Mix, and 2.0 µL DNA (10 ng/µL) in a 25 µL total volume. Cycling conditions were 95 °C for 3 s followed by annealing at 58 °C for 30 s, with fluorescence recorded at 72 °C during extension. Each run included two technical replicates, three biological replicates, No template control (NTC), and Positive control with the respective reference isolates. Data from two technical replicates were averaged and significance was observed using one-way ANOVA with Tuckey’s HSD (*p* < 0.05) using JMP (v8.0) (SAS Institute Inc., NC, USA). The Amplicon size was verified on 2% agarose gel, and the PCR products were sequenced at Genome Quebec to verify the genes.

##### Singleplex and Multiplex qPCR Assays

To optimize multiplex qPCR conditions, assays were first validated in singleplex runs, followed multiplex reactions including all four target genes. For singleplex assays, amplification was performed in a 25 µL reaction mixture containing: 1 μL of each primer (10 µM), 1 μL of each probe (10 µM), 2X Master Mix, and 2.0 µL DNA template (10 ng.µL^−1^). Cycling conditions were 95 °C for 3 s followed by annealing at 58 °C for 30 s, with fluorescence taken at 72 °C during extension. Multiplex assays were then performed in a 25 µL reaction mixture containing: 6 μL of primers (1 μL of each primer, 100 mM), 3 μL of probes (1 μL of each probe, 100 mM), 2X Master Mix, 2.5 μL of DNA template (20 ng/μL), and SDW to make up for the volume. Cycling conditions were identical to singleplex runs.

#### 5.6.3. Detection of Mycotoxins by Enzyme-Linked Immunosorbent Assay (ELISA)

Four mycotoxins (ochratoxins, aflatoxins, T2, and Deoxynivalenol) were quantified using AgraQuant^®^ ELISA kits: Ochratoxin A (2–40 ppb), Aflatoxin (4–40 ppb), T2 (20–500 ppb), and Deoxynivalenol (0.25–5 ppm). To reduce matrix effects from cannabis compounds, extracts were first purified using immunoaffinity columns (IAC): AFLAStar^®^, DONStar™, OCHRAStar^®^, and T2/HT2Star™.

For ochratoxin, aflatoxins, and T2 toxin, bud samples (0.5 g each, in triplicates, repeated twice) were extracted with 70% methanol at a 1:10 (*w*/*v*) ratio. For DON, samples were extracted with HPLC-grade water at a 1:5 (*w*/*v*) ratio. All assays were performed according to the manufacturer’s instructions. Briefly, 1 g of finely ground bud (2 mm particle size) was extracted with 70% methanol in a 1:5 (*w*/*v*) ratio, allowed to settle, and filtered through Whatman No. 1 paper. Aliquots of 100 µL of each extract were mixed with 200 µL of conjugate, and 100 µL of this mixture was applied to antibody-coated wells and incubated for 15 min. The wells were washed with sterile distilled water 5 times, followed by addition of 100 µL substrate to enhance color, and incubated for 5 min, and addition of 100 µL of stop solution. Color intensity was measured at 454 nm with a microplate reader.

Method performance for ochratoxins and DON was validated by spiking samples with pure standards, as described by [54]. Stock solutions of DON (100 µg/mL) and Ochratoxin (10 µg/mL) were prepared in methanol, diluted 1:10 with HPLC-grade methanol, and used for spiking. Cannabis samples (1 g) were spiked to final concentrations of 10, 20, and 30 ppb, passed through IAC columns, and analyzed as described above.

## Figures and Tables

**Table 1:** Overview of cannabis bud samples (IR, NR, and LP1-10) with cultivation method, chemotype, cannabinoid, terpenes, and lot information.

Sample	Cultivation	Chemotype	THC%	CBD%	Terpenes	Lot No.
NR ^1^	GH	N/A	N/A	N/A	N/A	2030
IR ^2^	GH	N/A	N/A	N/A	N/A	2030
LP1	H.GH	Sativa	15–21	0–1	Caryophyllene, Limonene, Myrcene	1104020000 579
LP2	H.GH	Indica	20–26	0–1	Caryophyllene, Humulene, Myrcene, Nerolidol, Pinene	AAA-112829
LP3	IN	Indica	12–16	0–1	Limonene, Terpinolene, Pinene	1001095
LP4	GH	Sativa	15–23	0–1	Alpha-Pinene, Alpha-Santalene, Beta-Caryophyllene, Myrcene, Selinadienes	HOU9232E0
LP5	IN	Sativa	15–18	0–1	Caryophyllene, Myrcene, Humulene	82333
LP6	GH	Hybrid	12–20	0–1	Caryophyllene, Humulene, Limonene, Linalool, Myrcene, Nerolidol, Pinene	3101719322
LP7	IN	Hybrid	15–20	0–1	Alpha-Pinene, Limonene, Myrcene, Terpinolene, Trans-Caryophyllene	JG01P-0031-A
LP8	IN	Indica	14–21	0–2	Alpha-Pinene, Beta-Caryophyllene, Beta-Myrcene, Beta-Pinene, Limonene	2002156
LP9	GH	Hybrid	17–23	0–0.5	Beta-Caryophyllene, Humulene, Limonene, Myrcene, Trans-Caryophyllene	7B3L1
LP10	H.GH	Hybrid	13–16	0–1	N/A	247

^1^ NR = non-irradiated sample; ^2^ IR = irradiated sample; LP = licensed producer sample; GH = greenhouse; H.GH = hybrid-greenhouse; IN = indoor; N/A = data not available.

**Table 2:** Summary of bacterial and fungal species identified in the samples.

Sample	CFU	Bacteria	CFU	Fungi
NR ^1^	7 × 10^3^	*Enterobacter hormaechei* *Pseudomonas aeruginosa*	5 × 10^4^	*A. flavus*, *A. japonicus*, *A. ochraceus*, *A. sclerotiorum*, *A. tamarii*, *F. proliferatum*, *C. oxysporum*, *P. copticola*, *P. citrinum*, *P. griseofulvum*
IR ^2^	0		3 × 10^4^	*A. flavus*, *C. tenuissimum*, *P. commune*, *P. copticola*, *P. citrinum*, *P. griseofulvum*
LP1	2 × 10^3^	*Methylobacterium tardum* *Pseudomonas aeruginosa*	0	N/A
LP2	0		0	N/A
LP3	0		0	N/A
LP4	0		0	N/A
LP5	2 × 10^3^	*Pseudomonas* sp. *Pseudomonas aeruginosa*	1.2 × 10^4^	*A. niger*, *A. tamarii*, *A. tubingensis*, *A. piperis*, *A. sydowii*, *A. awamori*, *A. chevalieri*, *C. globosum*, *P citrinum*, *P. commune*
LP6	0		0	
LP7	0		2 × 10^3^	*A. niger*, *P. citrinum*,
LP8	0		0	N/A
LP9	0		0	N/A
LP10	0		0	N/A

^1^ NR = non-irradiated sample; ^2^ IR = irradiated sample; LP = licensed producer sample; N/A = data not available.

**Table 3:** Overview of identified toxigenic fungal species isolated from the samples and the relevant reported mycotoxins.

Major Mycotoxins ^1^	Fungal Species	Health Hazards	References
AFB1/B2, AFG1/G2	*A. flavus*	Hepatotoxic, carcinogenic	[25,26,27]
OTA	*A. japonicus*, *A. ochraceus*, *A. sclerotiorum*	Genotoxic, immunosuppressive, teratogenic, mutagenic	[28,29]
FB1, FB2	*F. proliferatum*	Teratogenic, carcinogenic, neurotoxic	[30,31,32]
CPA	*A. tamarii*, *P. commune*	Nephrotoxic, hepatotoxic, neurotoxic	[33,34]
Roquefortine C	*P. commune*	Neurotoxic	[33,34]
Patulin	*P. copticola*, *P. griseofulvum*	Carcinogenic	[35,36,37]
Citrinin	*P. copticola*, *P. citrinum*	Nephrotoxic	[35]
Verruculogen	*P. simplicissimum*	Neurotoxic	[38,39]

^1^ Aflatoxins (AFB1, AFB2, AFG1, AFG2); Ochratoxin A (OTA); Fumonisins (FB1, FB2); Cyclopiazonic acid (CPA).

**Table 4:** Cq values and gene copy numbers for mycotoxin biosynthetic genes.

Sample	*Nor1*		*T5*		*Pks*		*DON*	
	Cq ^1^	Copies ^2^	Cq ^1^	Copies ^2^	Cq ^1^	Copies ^2^	Cq ^1^	Copies ^2^
NR	22.4 ± 0.2	8.0 × 10^7^	28.6 ± 0.1	4.83 × 10^8^	24.5 ± 0.4	2.11 × 10^9^	28.3 ± 0.4	2.07 × 10^11^
IR	22.6 ± 0.1	7.1 × 10^7^	28.7 ± 0.1	4.65 × 10^8^	24.9 ± 0.3	1.69 × 10^9^	29.6 ± 0.2	6.57 × 10^10^
LP1	0 + 00	0.0	36.7 ± 0.1	1.8 × 10^6^	31.3 ± 0.3	1.58 × 10^7^	35.7 ± 0.1	3.3 × 10^6^
LP2	35 ± 1	1.6 × 10^5^	36.5 ± 0.2	2.0 × 10^6^	27.9 ± 0.2	1.62 × 10^8^	35.2 ± 0.3	4.5 × 10^6^
LP3	35.8 ± 0.8	8.7 × 10^4^	34.8 ± 0.7	5.8 × 10^6^	26.4 ± 0.1	4.78 × 10^8^	34.7 ± 0.6	6.3 × 10^6^
LP4	35.0 ± 0.1	1.6 × 10^5^	0 + 00	0.0	26.5 ± 0.2	4.35 × 10^8^	34.7 ± 0.2	6.0 × 10^6^
LP5	35.3 ± 0.3	1.3 × 10^5^	35.2 ± 0.1	4.4 × 10^6^	32.2 ± 0.4	8.30 × 10^6^	34.4 ± 0.4	7.7 × 10^6^
LP6	34.8 ± 0.2	1.7 × 10^5^	34.5 ± 0.5	7.0 × 10^6^	27.0 ± 1	3.08 × 10^8^	32.8 ± 0.1	2.0 × 10^7^
LP7	35.3 ± 0.2	1.1 × 10^5^	35.2 ± 0.2	4.5 × 10^6^	25.9 ± 0.3	6.73 × 10^8^	33.3 ± 0.4	1.5 × 10^7^
LP8	35.0 ± 0.8	1.6 × 10^5^	37.8 ± 0.1	8.6 × 10^5^	26.9 ± 0.2	3.30 × 10^8^	32.0 ± 0.3	3.3 × 10^7^
LP9	0 + 00	0.0	0 + 00	0.0	0 + 00	0.0	33.8 ± 0.1	1.1 × 10^7^
LP10	35.3 ± 0.3	1.2 × 10^5^	0 + 00	0.0	31.4 ± 0.1	1.50 × 10^7^	32.1 ± 0.4	3.1 × 10^7^

^1^ Values represent mean Cq±SD and corresponding ^2^ gene copy numbers (copies. g^−1^ sample, *n* = 3). Statistical significance was assessed by one-way ANOVA followed by Tuckey’s HSD (*p* < 0.05).

**Table 5:** Analysis of ochratoxins, aflatoxins, deoxynivalenol, and T-2 mycotoxins in cannabis bud samples using ELISA antibody assay.

Sample	Ochratoxins	Aflatoxins	Deoxynivalenol	T2 Toxin
NR	8.1 ± 0.08	5.7 ± 1.09	2.08 ± 0.07	<LOD
IR	6.3 ± 0.7	4.9 ± 0.4	2.05 ± 0.5	<LOD
LP1	2.5 ± 0.12	<LOD	<LOD	<LOD
LP2	2.2 ± 0.2	<LOD	0.81 ± 0.2	<LOD
LP3	<LOD	<LOD	0.45 ± 0.0	<LOD
LP4	<LOD	<LOD	<LOD	<LOD
LP5	3.8 ± 0.9	<LOD	<LOD	<LOD
LP6	2.3 ± 0.4	<LOD	<LOD	<LOD
LP7	2.2 ± 0.2	<LOD	<LOD	<LOD
LP8	3.6 ± 0.4	<LOD	<LOD	<LOD
LP9	2.8 ± 0.3	<LOD	<LOD	21 ± 0.5
LP10	2.5 ± 0.1	<LOD	<LOD	23.8 ± 0.2

Values represent mean mycotoxin concentration ±SD. Statistical significance was assessed by one-way ANOVA followed by Tuckey’s HSD (*p* < 0.05). LOD-limit of detection, Detection ranges of the ELISA kits: ochratoxins (2–40 ppb); Aflatoxins (4–40 ppb); Deoxynivalenol (0.25–5 ppm); T2 toxins (20–500 ppb).

**Table 6:** qPCR primers and probes designed and used in this study.

Toxin ^1^	Primer	Sequence	Amplicon	References
Aflatoxin	Nor1-F	5′-ATGTATGCTCCCGTCCTACT-3′	396	This study
(AY371490.1)	Nor1-R	5′-ATGTTGGTGATGGTGCTGAT-3′		
	Probe	5′ FAM-ACAAACTTGGCCTGTTGCTTGGAC-IB 3′		
Trichodiene	Tri5-F	5′-TCTTAACACTAGCGTGCGCCTTCT-3′	193	[53]
(AF336366)	Tri5-R	5′-CATGCCAACGATTGTTTGGAGGGA-3′		
	Probe	5′ HEX-AACAAGGCTGCCCACCACTTTGCTCAGCCT-IB 3′		
Deoxynivalenol	Tri13-F	5′-CTTGTGCGAGTTTGGGTATTG-3′	282	This study
(AY064209)	Tri13-R	5′-AGTGTACTCAGCATCCGATATG-3′		
	Probe	5′ Cy3-CCTGGGTTGGAAGGAATGGAGACC-IB 3′		
Ochratoxin	Pks-F	5′-AGTGATGACTGGAGGGAGGTGAAT-3′	199	[53]
(AY320069)	Pks-R	5′-ACGAGCATGCGGTATCAATGGTCA-3′		
	Probe	5′ TR-TTGTCCGGCAGGATCAGGTGCCCACCATT-IB 3′		

^1^ Target toxin and GenBank sequence.

## Data Availability

The original contributions presented in this study are included in the article/Supplementary Materials. Further inquiries can be directed to the corresponding authors.

## Supplementary Materials

The following supporting information can be downloaded at: https://www.mdpi.com/article/10.3390/toxins17110528/s1, Figure S1: Representative images of fungal growth of irradiated (IR) and non-irradiated (NR) cannabis samples on different media (10^−3^ dilution). MEA = malt extract agar; PDA = potato dextrose agar; LBA = Luria–Bertani agar; DRBC = dichloran–rose Bengal–chloramphenicol agar. Figure S2. Representative images of fungal growth of licensed produced cannabis samples (LP1, 5, 7) on PDA and DRBC media (direct plating). PDA = potato dextrose agar; DRBC = dichloran–rose Bengal–chloramphenicol agar. Table S1. CFU counts and microbial taxa isolated from NR, IR, and LP1-10 cannabis samples on selective and non-selective media. Table S2. Relative abundance of fungal genera detected by NGS in cannabis samples. Table S3. PCR primers designed and used in this study.
