# Supplementary material for: Detection of Mycotoxigenic Fungi and Residual Mycotoxins in Cannabis Buds Following Gamma Irradiation
Source: Toxins (Basel). 2025 Oct 28;17(11):528. doi: 10.3390/toxins17110528 (PMC12656284; doi:10.3390/toxins17110528)
Supplement: Supplementary file 1 [file toxins-17-00528-s001.zip › toxins-3919308-supplementary.pdf]

toxins

Article

Detection of Mycotoxigenic Fungi and Residual Mycotoxins in Cannabis Buds Following Gamma Irradiation

Mamta Rani [1,*,†], Mohammad Jamil Kaddoura [1,†], Jamil Samsatly [2], Guy Chamberland [2], Suha Jabaji [1] and Saji George [1,*]

[1] Department of Food Science and Agricultural Chemistry, McGill University, Montreal, QC H9X 3V9, Canada; mohammad.kaddoura@mail.mcgill.ca (M.J.K.); suha.jabaji@mcgill.ca (S.J.)
[2] BioSun Solutions, Biotechnology Company, Chambly, QC J3L 4V2, Canada; jsamsatly@biosunsolutions.com (J.S.); guy-chamberland-masterherbalist@live.ca (G.C.)
* Correspondence: mamta.rani@mcgill.ca (M.R.); saji.george@mcgill.ca (S.G.)
† These authors contributed equally to this work.

1. Supplementary

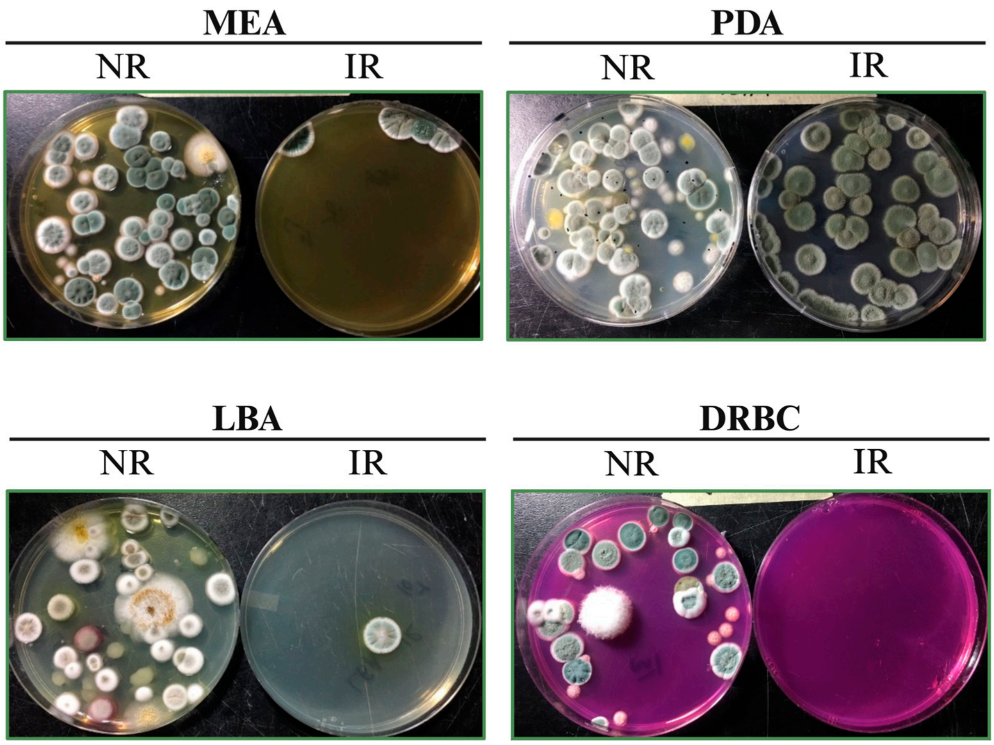

Figure S1. Representative images of fungal growth of irradiated (IR) and non-irradiated (NR) cannabis samples on different media (10^{-3} dilution). MEA = malt extract agar; PDA = potato dextrose agar; LBA = Luria–Bertani agar; DRBC = dichloran–rose Bengal–chloramphenicol agar.

 https://doi.org/10.3390/xxxxx

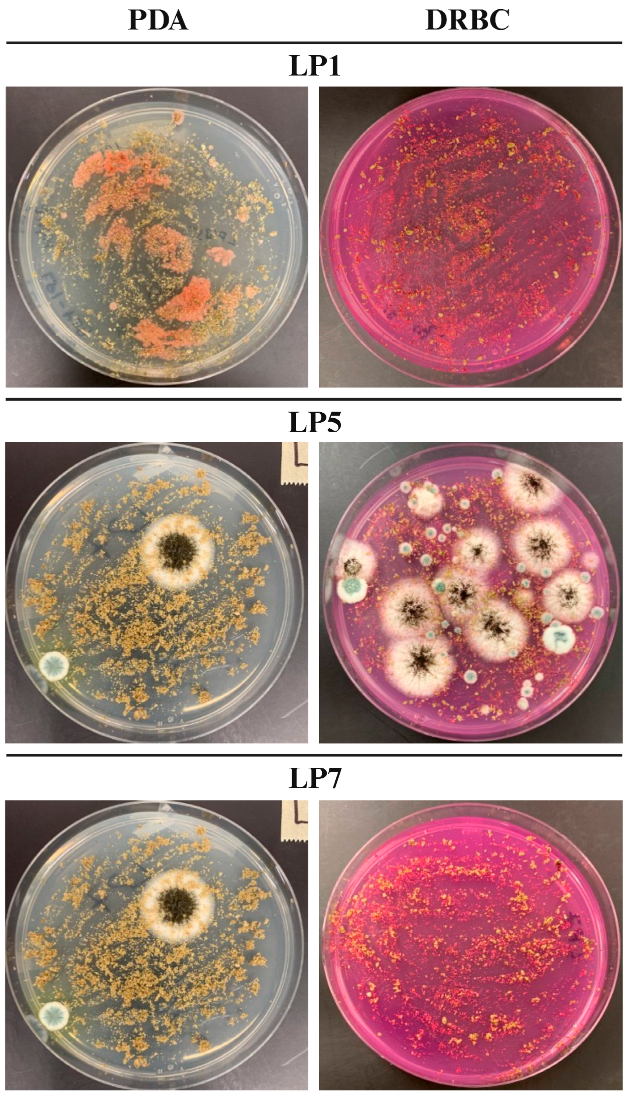

Figure S2. Representative images of fungal growth of licensed produced cannabis samples (LP1, 5, 7) on PDA and DRBC media (direct plating). PDA = potato dextrose agar; DRBC = dichloran–rose Bengal–chloramphenicol agar.

Table S1. CFU counts and microbial taxa isolated from NR, IR, and LP1-10 cannabis samples on selective and non-selective media.

Sample[1]	Media[2]	Bacteria CFU[3]	Bacteria Species	Fungi CFU[3]	Fungi Species
NR	MEA	0	-	$5x10^4$	*A. flavus, A. japonicus, A. ochraceus, A. sclerotiorum*
	LBA	$2x10^3$	*E. hormaechei, P. aeruginosa*	$2x10^4$	*A. japonicus, C. oxysporum, A. tamarii, F. proliferatum, P. copticola*
	PDA	$7x10^3$	*E. hormaechei, P. aeruginosa*	$5x10^4$	*Penicillium sp., P. citrinum*
	DRBC	0	-	$3x10^4$	*P. simplicissimum, A. flavus, A. japonicus, A. ochraceus*
	TSA	0	-	0	-
IR	MEA	0	-	$4x10^3$	*A. flavus, C. tenuissimum, P. commune*
	LBA	0	-	$1x10^3$	*P. copticola, P. citrinum, P. griseofulvum*
	PDA	0	-	$3x10^4$	*A. flavus, P. commune*
	DRBC	0	-	0	-
	TSA	0	-	0	-
LP1	PDA	2 x103	*M. tardum, P. aueroginosa*	0	-
	LBA	0	-	0	-
	DRBC	0	-	0	-
LP2	PDA	0	-	0	-
	LBA	0	-	0	-
	DRBC	0	-	0	-
LP3	PDA	0	-	0	-
	LBA	0	-	0	-
	DRBC	0	-	0	-
LP4	PDA	0	-	0	-
	LBA	0	-	0	-
	DRBC	0	-	0	-
LP5	PDA	$2 x10^3$	*Pseudomonas sp.*	$1.1x10^4$	*A. niger, A. tamarii, A. tubingensis, A. piperis, A. sydowii, A.awamori, A.chevalieri, C. globosum, P. citrinum, P. commune*
	LBA	$2 x10^3$	*P. aueroginosabacteri*	$1.2x10^4$	*A. niger, A. tamarii, A. tubingensis, A. piperis, A. sydowii, A.awamori, A.chevalieri, C. globosum, P. citrinum, P. commune*
	DRBC	0	-	$8x10^3$	*A. niger, P. citrinum*
LP6	PDA	0	-	0	-
	LBA	0	-	0	-
	DRBC	0	-	0	-
LP7	PDA	0	-	$2 x10^3$	Penicillium citrinum, Aspergillus niger
	LBA	0	-	0	-
	DRBC	0	-	0	-
LP8	PDA	0	-	0	-
	LBA	0	-	0	-
	DRBC	0	-	0	-
LP9	PDA	0	-	0	-
	LBA	0	-	0	-
	DRBC	0	-	0	-
LP10	PDA	0	-	0	-
	LBA	0	-	0	-
LP10	DRBC	0	-	0	-

[1] NR = non-irradiated sample; IR = irradiated sample; LP = licensed producer sample; [2] CFU = Colony forming units (CFU.g^{-1}); [3] MEA = Malt extract agar; LBA = Luria-*Bertani* Agar, PDA = Potato dextrose agar, TSA = Tryptic Soy Agar, DRBC = Dichloran-rose Bengal-chloramphenicol agar.

Table S2. Relative abundance of fungal genera detected by NGS in cannabis samples.

Genus	NR	IR
Penicillium copticola	0.00284	8.99E-05
Penicillium simplicissimum	0.00122	0.00025
Diaporthe caulivora	0.00063	2.40E-05
Conocybe nigrescens	0	0.00032
Penicillium steckii	8.99E-05	0.00019
Penicillium olsonii	8.39E-05	0.0001
Sarocladium strictum	1.20E-05	8.99E-05
Cladosporium sphaerospermum	7.79E-05	3.60E-05
Aspergillus ochraceus	6.59E-05	1.20E-05
Trichoderma hamatum	5.99E-06	6.59E-05
Others	0.99573	0.99883

Table S3. PCR primers designed and used in this study.

Target	Primer	Sequence	Amplicon	References
Aflatoxin	Nor1-F	5′-ACCGCTACGCCGGCACTCTCGG-3′	396	[56]
	Nor1-R	5′-GGCCGCCAGCTTCGACACTCCG-3′		
Trichothecene family	Tri6-F	5′-GATCTAAACGACTATGAATCACC-3′	541	[57]
	Tri6-R	5′-GCCTATAGTGATCTCGCATGT-3′		
Trichodiene	Tri5-F	5′-TCTTAACACTAGCGTGCGCCTTCT-3′	193	[58]
	Tri5-F	5′-CATGCCAACGATTGTTTGGAGGGA-3′		
Deoxynivalenol	Tri13-F	5′-CATCATGAGACTTGTKCRAGTTTGGG-3′	282	[59]
	Tri13-F	5′-GCTAGATCGATTGTTGCATTGAG-3′		
Ochratoxin	Pks-F	5′-AGTGATGACTGGAGGGAGGTGAAT-3′	199	[58]
	Pks-R	5′-ACGAGCATGCGGTATCAATGGTCA-3′		
